# Biliary atresia in preterm infants: a single center experience and review of literature

**DOI:** 10.3389/fsurg.2024.1353424

**Published:** 2024-03-01

**Authors:** Federico Beati, Antonella Mosca, Andrea Pietrobattista, Daniela Liccardo, Sara Ronci, Lidia Monti, Paola Francalanci, Marco Spada, Giuseppe Maggiore, Pietro Bagolan, Fabio Fusaro

**Affiliations:** ^1^ Neonatal Surgery Unit, Area of Fetal, Neonatal and Cardiological Sciences, Bambino Gesù Children’s Hospital, IRCCS, Rome, Italy; ^2^ Hepatogastroenterology and Liver Transplant Unit and Medical Genetics Laboratory, Bambino Gesù Children’s Hospital, IRCCS, Rome, Italy; ^3^ Department of Radiology, Bambino Gesù Children’s Hospital, IRCCS, Rome, Italy; ^4^ Pathology Unit, Department of Diagnostic and Laboratory Medicine, Bambino Gesù Children’s Hospital, IRCCS, Rome, Italy; ^5^ Division of Abdominal Transplantation and Hepato-Bilio-Pancreatic Surgery Unit, Bambino Gesù Children’s Hospital, IRCCS, Rome, Italy; ^6^ Department of Systems Medicine, University of Tor Vergata, Rome, Italy

**Keywords:** biliary atresia—common cause of obstructive jaundice in young infants, Kasai portoenterostomy (KPE), clearance of jaundice, neonatal cholestasis, pediatric liver transplant, native liver survival

## Abstract

**Introduction:**

The diagnosis of biliary atresia (BA) remains challenging, and there is still uncertainty regarding the optimal time to perform a Kasai portoenterostomy (KPE). Little is known about the difficulties in the diagnosis and outcomes of BA in preterm infants (PBA). This study, which represents the first Italian report of preterm infants with BA, aims to describe a single-center experience of BA in preterm newborns.

**Methods:**

We retrospectively reviewed all infants consecutively diagnosed with BA who underwent a Kasai procedure at the Bambino Gesù Children’s Hospital between January 1998 and December 2021. Prematurity was defined as a gestational age (GA) of <37 weeks. Demographic, laboratory, and histology data were recorded, and the main outcomes considered were clearance of jaundice (COJ), native liver survival, and mortality.

**Results:**

A total of 21 PBA were compared with 117 term BA controls (TBA). The median GA of PBA was 35.1 (32–36.1) weeks, with a mean birth weight of 2,100 (1,897–2,800) g. Age at first presentation was significantly lower in PBA patients: 46 (22–68) vs. 61 (44–72) days; *p *= 0.02. The median age at KPE was similar between the two groups: 70 days (33 corrected) for PBA vs. 67 in TBA; *p *= 0.8. At the time of surgery, median serum bilirubin was lower in the PBA group (7.7 vs. 8.6 mg/dl, *p* = 0.04). Similarly, the median APRi at the time of KPE was lower but not significant in the PBA group: 1.09 vs. 1.16; *p *= 0.8. No differences were found in terms of COJ between the PBA and TBA groups: *n *= 9 (43%) vs. 34 (35%); *p *= 0.2. Overall native liver survival was similar between the two groups: 8.6 (4.8–12.2) for the PBA group vs. 7.6 (5.6–9.5) years for the TBA group with no significant differences; *p *= 0.45. Post-KPE native liver survival was similar between the two groups: 38% vs. 52% at 5 years for the TBA and PBA groups, respectively;   *p *= 0.54.

**Conclusion:**

The PBA and TBA groups appear to have similar outcomes in terms of COJ, overall native liver survival, and 5-year liver survival. Considering the corrected GA, early KPE is related to lower cholestatic damage. Further multicenter studies are required.

## Introduction

Biliary atresia (BA) is a rare, idiopathic, progressive disease affecting the biliary tract that can lead to end-stage cirrhosis and liver failure if left untreated. BA continues to be the principal indication worldwide for pediatric liver transplantation (LT) (1–3). It represents approximately 25%–30% of neonatal cholestasis (NC) cases, with a worldwide incidence rate ranging from 1:5,000 to 1:18,000 (4–7); no Italian epidemiological data have been reported over the years. Kasai portoenterostomy (KPE) has dramatically improved the outcome of these patients; however, many factors may influence the postoperative outcomes, including age at surgery, the extent of liver damage, the surgical experience of the center, and adjuvant therapies (8–10). Prematurity has been reported as a risk factor for BA, although the exact mechanism remains unclear and data on the clinical condition and outcome of preterm infants suffering from BA are limited. Some authors reported a higher incidence of BA among preterm infants (PBA) (1.06/10,000) compared with full-term infants (0.52/10,000) (11). In premature babies, the diagnosis may be difficult due to many confounding factors such as the use of parenteral nutrition (PN) during intensive neonatal care, lack of minimal enteral feeding, prematurity in biliary enterohepatic circulation, and recurrent sepsis (12, 13). Furthermore, infants delivered prematurely may be prone to bile duct damage when out of the *in utero* environment (14). These diagnostic challenges might result in delayed referral, treatment, and, subsequently, worse outcomes.

The aim of this study, which represents the first Italian report of preterm infants with BA, is to describe a single-center experience.

## Materials and methods

We performed an observational (cross-sectional) retrospective chart review of all infants consecutively diagnosed with BA at the Bambino Gesù Children's Hospital between January 1998 and December 2021. Patients who underwent primary LT. All data were retrospectively collected and recorded according to the Declaration of Helsinki.

Data about clinical, laboratory, and radiological presentation together with the outcomes of PBA were compared with those of the full-term BA infants (TBA). Prematurity was defined as a gestational age (GA) of <37 weeks according to the WHO and categorized as “extremely” (<28 weeks of GA), “very” (28–32 weeks of GA), and “moderate to late” (33–36 weeks of GA) preterm. The corrected ages were calculated by subtracting the number of weeks of prematurity from the chronological age.

The collected data include GA, birth weight (BW), sex, age at the first presentation at our hospital defined as primary consult of pediatric hepatologist/pediatric surgeon (chronological age and corrected age for PBA), age at KPE (chronological age and corrected age for PBA), bilirubin and aspartate aminotransferase/platelet ratio (APRi) at KPE, diagnosis delay (defined as the time from the first presentation in our center to KPE), length of hospital stay (LoS), and postoperative LoS (days from surgery to discharge).

In infants with NC, the primary aim was to rule out/recognize those with BA. Patients with pale (hypocholic) stools and a liver ultrasound with specific signs of BA rapidly underwent liver biopsies or intraoperative cholangiography to rule out BA after exclusion of alpha-1 antitrypsin (A1AT) deficiency by serum enzymatic activity and cystic fibrosis by sweat test or genetic analysis. Conversely, all infants without high suspicion of BA at presentation underwent a full NC diagnostic work-up according to the guidelines available at the time of their presentation (15–23). First-line microbiology screening included CMV, TORCH, and HSV-1/2 serology.

All patients underwent screening for associated malformations, including brain ultrasound, urinary system ultrasound, cardiological evaluation, and echocardiogram.

Once the clinical suspicion of BA was confirmed, the patients were divided into three categories depending on the congenital anomalies found:

- BA associated with splenic malformation (BASM)
- BA associated with cardiac anomalies
- BA associated with other isolated congenital malformations

BASM syndrome was defined by the presence of typical anomalies, such as polysplenia/absent spleen, situs inversus, preduodenal portal veins, cardiac anomalies, and the absence of the vena cava (24).

### Surgery

**Figure 1:**
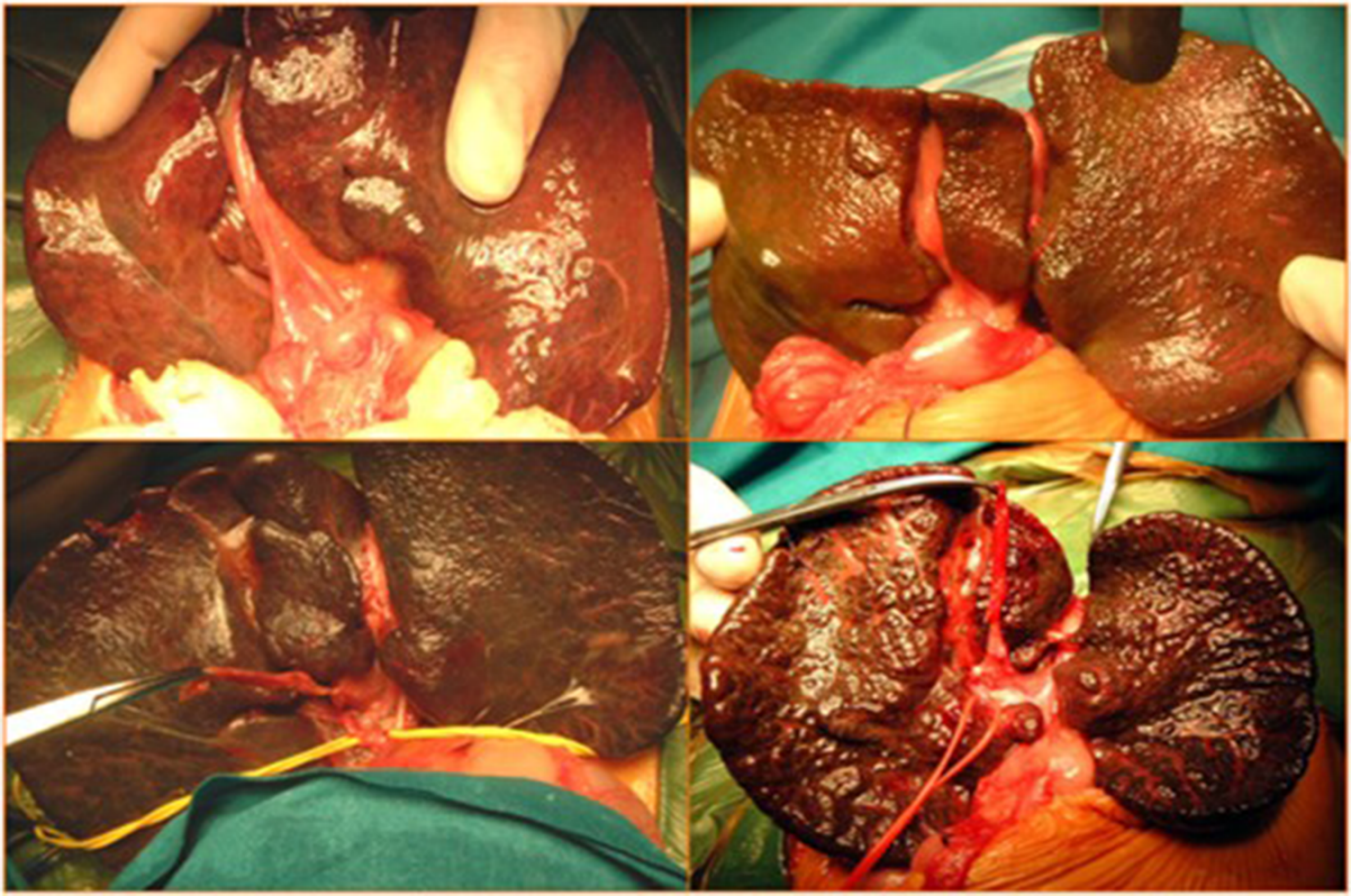
Intraoperative images of Kasai portoenterostomy (KPE).

All patients with confirmed diagnoses of BA underwent KPE at our hospital. KPE was defined as “delayed” when performed after 60 days of life and “very delayed” when performed after 90 days of life (Figure 1).

All KPE procedures were performed by surgeons in a high-volume liver surgery unit, and anestesiological procedures were performed by dedicated neonatal anesthesiologists.

### Histology

At the time of KPE, liver biopsy at the portal region was performed in all patients and analyzed retrospectively by a single group of pathologists. Liver fibrosis was classified based on the Ishak score (25). Ductal plate malformation (DPM) was considered to be present by the presence of specific alterations (26–28).

### Postoperative protocol

All patients received postoperative clinical treatment according to the protocol enforced in our hospital.

More specifically, from 1999 to 2015, adjuvant steroid therapy and antibiotic prophylaxis included the following:

1. Antibiotics: intravenous antibiotics—100 mg/kg/day of ceftriaxone for 5 days plus 7.5 mg/kg/day of amikacin for 2 days.
2. Steroids:
  a. Day 1: 10 mg/kg/day of IV methylprednisolone, which is decreased by 2 mg every 24 h until a dose of 2 mg/kg/day and then switched with oral administration.
  b. Day 6: 2 mg/kg/day of oral prednisolone for 15 days.
  c. Day 21: 1 mg/kg/day of oral prednisolone for 15 days.
  d. Day 36: 0.5 mg/kg/day of oral prednisolone for 15 days.
  e. Day 50: 0.25 mg/kg/day of oral prednisolone for 15 days and then discontinued
3. Long-term oral antibiotic prophylaxis should be started on the 6th postoperative day, with cycles of co-amoxiclav (25 mg/kg/day) and trimethoprim (5 mg/kg/day); each cycle of 3 weeks with a 1-week break between cycles for a total of 12 months after surgery.
4. Other drugs: ranitidine and fat-soluble vitamins.
5. Ursodeoxycholic acid that was already started before KPE at a dose of 10 mg/kg/day was continued during follow-up, adapting the dose to the weight of the patients.

From 2016 to 2021, the protocol was modified, eliminating the administration of adjuvant steroid therapy and long-term oral antibiotic prophylaxis with no differences in terms of clearance of jaundice (COJ) and native liver survival, as previously published (10).

### Follow-up

All patients were included in a long-term single-center follow-up. Outpatient visits were scheduled according to a standard protocol within 1 week, 1 month, and every 3 months up to 1 year after surgery. Subsequent visits were scheduled based on clinical conditions, laboratory, and radiological results, at least annually.

Follow-ups were considered from surgery to the last outpatient visit included in the study period or to the last evaluation before LT or death.

### Outcomes

The main outcomes considered were COJ, defined as total serum bilirubin (BST) of <1.17 mg/dl within 6 months after surgery, 5-year native liver survival, overall native liver survival (defined as the time between KPE and LT or the last follow-up), and mortality. All data obtained from the TBA and PBA groups were compared.

### Statistics

Data were analyzed using the MedCalc Statistical Software Version 20.216 (MedCalc Software Ltd., Ostend, Belgium; https://www.medcalc.org; 2023). Both groups were compared using the Fisher's exact test and *χ*² test for trend as appropriate. The results are reported as median and interquartile range (IQR), and *p* < 0.05 (two-sided) was considered statistically significant. The unpaired *T* test or Mann–Whitney *U* test were used for univariate analysis, if applicable. Spearman-rank correlation was used to measure correlation. Native liver survival was illustrated by Kaplan–Meier survival curves, and the differences were assessed using a log-rank test. *P* < 0.05 was considered significant.

## Results

### Demographic data

A total of 155 patients were collected from our monocentric register of BA. Among them, we excluded 17 patients who underwent primary LT because of advanced age at presentation and/or liver failure. They were all full-term born infants with non-syndromic BA.

A total of 138 patients were thus included in this study. All of them had KPE as the primary procedure, which was performed in our hospital. Among them, 21 PBA (9 males, 12 females) were compared with the 117 term TBA newborns (42 males, 75 females).

The demographic data are shown in Table 1.

**Table 1:** Demographic data of study population.

	Term (117)	Preterm (21)	*p*
GA (weeks)	39, 4 (38.1–40.6)	35, 1 (32–36.1)	0.04
GA 27, <32 weeks, *n* (%)	\	2 (10)	
GA 32, <37 weeks, *n* (%)	\	19 (90)	
Birth weight (g)	3,200 (2,975–3,450)	2,100 (1,897–2,800)	0.01
Male/female, *n* (%)	42/75 (36/64)	9/12 (43/57)	

### Clinical parameters and laboratory and histological pictures

Age at first presentation was significantly lower in PBA patients: 46 (22–68) vs. 61 (44–72) days.

The median age at KPE was similar in the two groups: 67 (52–81) days for the TBA group and 70 (45–76) days for the PBA group; *p* = 0.8. Considering the corrected GA, the median age at KPE was 33 (16.7–47.2) days for the PBA group.

At the time of surgery, median serum bilirubin was lower in the PBA group (7.7 vs. 8.6 mg/dl, *p* = 0.04). Similarly, median APRi at the time of KPE was lower but not significant in the PBA group: 1.09 (0.6–2) vs. 1.16 (0.7–1.5); *p* = 0.8.

Among the preterm group, 10 patients (47%) had a delayed KPE (>60 days), and 2 patients (4%) had a very delayed KPE (>90 days). However, considering the corrected age, only one preterm infant had a KPE at >60 days.

The median Ishak fibrosis score from liver biopsies at the time of KPE was significantly lower in the PBA group compared with that in the TBA group (3.89 vs. 4.6, *p* = 0.001).

No differences in adjuvant corticosteroid use were seen between the two groups: 71/117 (60%) vs. 13/21 (61%), *p* = 0.7.

Specific data are shown in Table 2.

**Table 2:** Patient characteristics of preterm biliary atresia (PBA) compared with term biliary atresia (TBA) groups.

	Term (117)	Preterm (21)	*p*
Age at first presentation (days)	61 (44–72)	46 (22–68)	**0.02**
Corrected age at presentation		23 (3–39)	NA
Age at KPE (days)	67 (52−81)	70 (45–76)	0.8
Corrected age at KPE (days)		33 (16.7–47.2)	NA
Bilirubin levels at KPE (mg/dl)	8.6 (7.3–10.4)	7.7 (6–8.8)	**0.04**
APRi at KPE	1.16 (0.7–1.5)	1.09 (0.6–2)	0.8
Kasai > 60 days *n* (%)	59 (50)	12 (42)	0.6
Ishak score at KPE	4.6 (3–6)	3.89 (2–6)	**0.001**
DPM + *n* (%)	30 (34)	6 (33)	0.9
Post-KPE adjuvant steroids treatment *n* (%)	71 (60)	13 (61)	0.7
Diagnosis delay (days)	8 (6–15.2)	8.5 (4–21)	0.76
Length of hospital stay (days)	20 (15–29)	22 (15–46)	0.15
Postoperative hospital stay (days)	13 (8–16)	13 (9.5–32)	0.39

Data are reported as median [interquartile range (IQR)].

The bold values mean statistically significant results.

Isolated cardiac anomalies were identified with a higher incidence in preterm patients than that in the term group (6/21 vs. 9/117; *p* = 0.03). Among the PBA group, only one patient underwent cardiac surgery for double aortic-arch correction after 6 months from KPE.

A team of cardiothoracic anesthesiologists was involved during the anesthesiological procedures for patients with major cardiac malformations.

Details on congenital anomalies are shown in Table 3.

**Table 3:** Associated anomalies.

	Term (117)	Preterm (21)	*p*
BASM	**14 (12%)**	**1 (5%)**	**0.4**
Isolated cardiac anomalies	**9 (7%)**	**6 (21%)**	**0.03**
Fallot tetralogy	0	1	
ASD	7	3	
VSD	0	1	
Double aortic-arch	0	1	
CAV	1	0	
Aortic valve stenosis	1	0	
Others anomalies	**8 (7%)**	**3 (14%)**	**0.5**
Duodenal atresia	0	1	** **
Ileal atresia	1	0	
Malrotation/PDPV	3	1	
Kabuki syndrome	1	0	
Genitourinary	1	1	
Neurology	2	0	
Endocrinology	0	1	

BASM, biliary atresia splenic malformation; PDPV, preduodenal portal vein; ASD, atrial septal defect; VSD, ventricular septal defect; AVC, atrioventricular canal.

The bold values mean statistically significant results.

### COJ

Among the TBA group, 18 patients (15%) underwent LT within 6 months of KPE and were, therefore, not evaluated for this outcome.

No differences were found in terms of COJ between the PBA and TBA groups: *n* = 9 (43%) vs. 34 (35%), *p* = 0.2.

**Figure 2:**
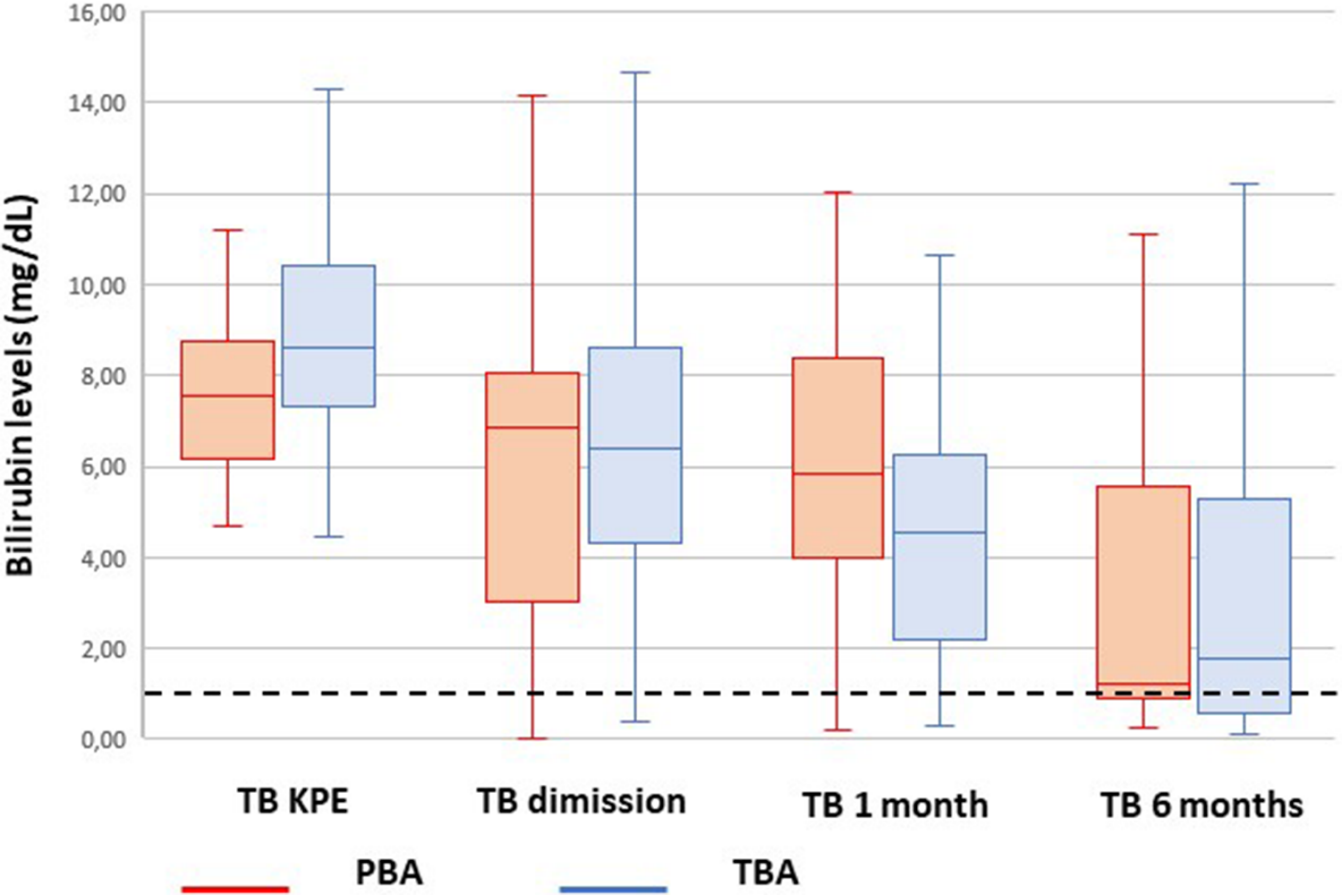
Trend of bilirubin from Kasai portoenterostomy (KPE) to 6 months. The median values are reported as horizontal lines inside each box plot. The dotted line shows the threshold for jaundice clearance (<1.17 mg/dl).

The bilirubin trend before and after KPE is shown in Figure 2.

### Liver transplant

A total of 70 patients in the TBA group (62%) and 11 patients in the PBA group (52%) underwent LT during the follow-up period considered.

In 12 patients from the TBA group, it was not possible to have clinical information regarding the outcome considered: among them, 6 (50%) patients were referred to adult centers for hepatological follow-up, 3 (25%) patients died after the KPE procedure, an additional 3 (25%) patients were lost to follow-up.

Regarding the PBA group, two (9%) patients were lost to follow-up.

**Figure 3:**
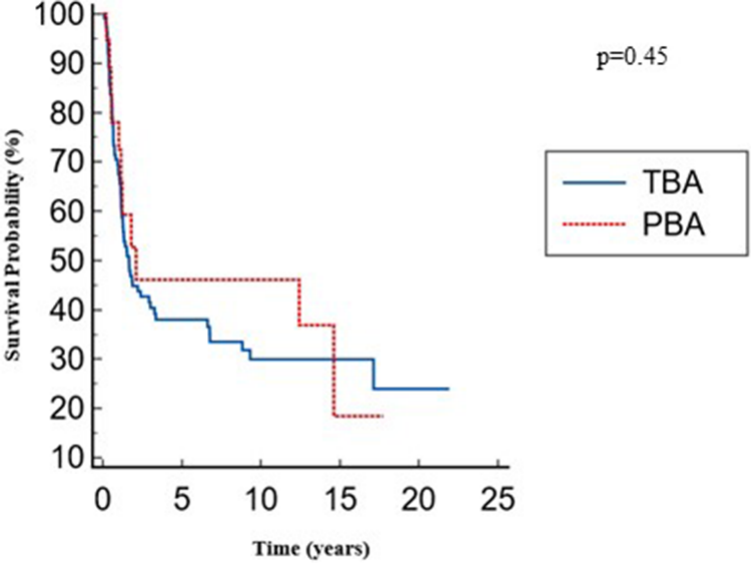
Overall native liver survival.

The overall native liver survival was similar between the two groups: 8.6 (4.8–12.2) for the PBA group vs. 7.6 (5.6–9.5) years for the TBA group with no significant differences; *p* = 0.45 (Figure 3).

**Figure 4:**
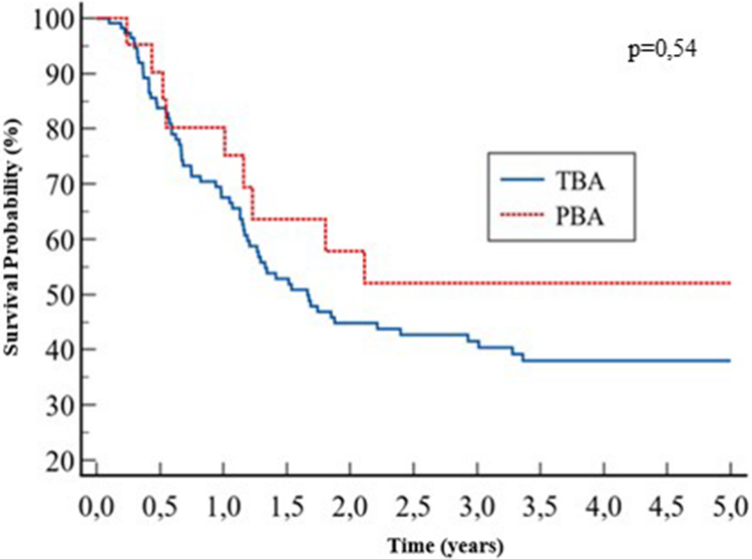
Kaplan–Meier of native liver survival at 5 years.

Post-KPE native liver survival was similar between the two groups: 38% vs. 52% at 5 years, for the TBA and PBA groups, respectively; *p* = 0.54 (Figure 4).

### Overall survival

The median follow-up was 1.2 (0.5–12.6) years. The mortality rate after KPE for the PBA group was 1/21 (4.7%) vs. 3/117 (2%) for the TBA group with no statistically significant difference (*p* = 0.7). One PBA patient died due to septic shock and multiple organ failure following a dehiscence of the porto-entero-anastomosis. He was born at 33 weeks of EG (BW, 1,600 g) with tetralogy of Fallot.

Among the TBA group, three patients died several months after KPE. One patient was affected by the Kabuki syndrome characterized by a severe immunological defect. She died 8 months after KPE for a multiorgan failure due to a septic shock. The other patient was affected by a polymalformative syndrome characterized by ambiguous genitalia, persistent polyuria, and bronchodysplasia. He died 7 months after KPE because of severe respiratory distress and septic shock, due to a central venous catheter-related infection. The third patient died 4 months after KPE in another center due to septic shock, but there was no more information about it.

### Correlations

**Figure 5:**
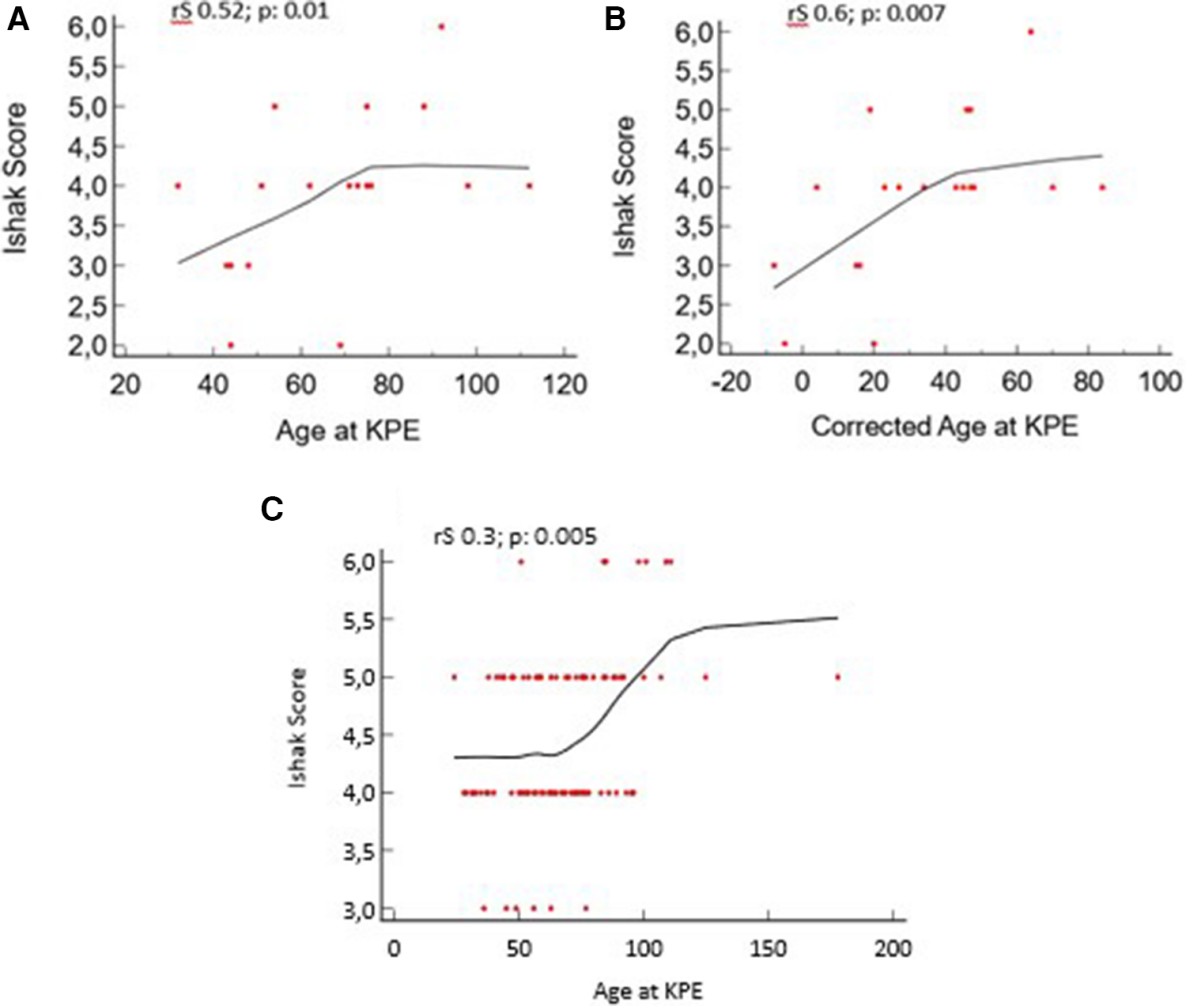
Liver fibrosis vs. age at Kasai portoenterostomy (KPE) and corrected age at KPE for preterm biliary atresia (PBA) group. (**A**, **B**) Age and corrected age for the PBA group at KPE. (**C**) Age for the term biliary atresia (TBA) group at KPE.

We found a positive correlation between the postnatal age and corrected age at KPE with the Ishak score in the PBA group: rS 0.52, *p* = 0.01, and rS 0.6, *p* = 0.007, respectively. A positive correlation is also present in the TBA group: rS 0.31, *p* = 0.005 (Figure 5).

We analyzed the presence of possible confounding factors for the diagnosis of BA, such as total PN and sepsis. Among the 12 preterm patients who received “delayed” KPE, only one (8%) patient received TPN before surgery, and only one (8%) patient was diagnosed with sepsis. TPN and sepsis, therefore, did not influence delayed KPE because there was no significant difference for TPN and sepsis (*p* = 0.26 and 0.98, respectively).

when comparing patients who received early KPE with patients who underwent delayed/very delayed KPE.

## Discussion

Today, there are only a few international nationwide studies that focus on this rare association, all describing poor outcomes in terms of COJ and native liver survival (11, 13, 29).

Van Wessel et al. in 2017 analyzed the outcomes of preterm patients with BA. They reported BA incidence among premature patients of 1.06/10,000 live births, almost double than that of the full-term born patients (0.54/100,000 live births), suggesting increased attention in the early diagnosis of AVB in the premature patients with cholestatic jaundice and acholic stools.

They also described poor outcomes in terms of native liver survival at 4 years from KPE: 21% in the premature group vs. 43% in the term group.

Similarly, Chiu et al. analyzed 27 preterm patients compared with 170 term patients diagnosed with BA over a time period from 2004 to 2007.

They reported a higher incidence of congenital anomalies among preterm patients (18.5% vs. 4.1%) and delayed surgery (71.8 vs. 52.9 days) compared with full-term patients.

In addition, a lower COJ rate at 6 months from KPE (37% vs. 62%) was reported for preterm patients and a lower native liver survival rate at 18 months after KPE (50% vs. 72%), advancing the hypothesis that a lower success rate of KPE might be caused by a higher sensitivity of the immature liver to cholestasis and associated inflammation (29).

Conversely, Durkin et al. described a similar and even better outcome in preterm newborns compared with full-term patients with BA. They first advanced the hypothesis of a protective role given by prematurity to liver damage caused by cholestasis (31).

According to the most recent ESPGHAN and NASPGHAN guidelines, despite BA being the most common cause of infantile cholestasis, late diagnosis remains a problem worldwide (19), and prematurity does not seem to influence the time of BA diagnosis but rather seems to be responsible for delayed surgery (30).

Our data showed an earlier evaluation of preterm infants in our tertiary level center compared with full-term babies (46 vs. 61 days, respectively). In this view, prematurity could represent a key factor for early referral. Moreover, our results showed a comparable timing of diagnosis among the two groups.

Durkin et al. (31) reported a higher but not significant use of PN in patients diagnosed after 50 days. This finding could be explained by the slightly lower GA of that series compared with our patients. Some preterm infants may present transient multifactorial cholestasis with hypocholic stools and histology showing bile duct proliferation, resembling BA. Despite these confounding factors that can hamper BA diagnosis during NC work-up, all the guidelines strongly recommend the same diagnostic process for NC regardless of GA, namely, a measurement of conjugated serum bilirubin in preterm infants with jaundice after 2 weeks of age (19).

In our series, there were no delays in terms of diagnosis and surgery in preterm patients, with a median age at KPE of 70 days (33 days considering corrected age); moreover, KPE was performed at an age of ≤90 days in all patients, defined in the literature as the optimal age (11, 29, 30, 32).

In our cohort of 117 full-term patients, the median age at KPE was 67 days, which is not different from PBA. Among them, nine patients (7%) were referred at a mean age of 115 days of life thus resulting in a “delayed” KPE. This rate of late referrals observed in our TBA patients underlines the absence of a national centralization policy on BA management, which overlooks the observation of these patients predicting worse outcomes (32).

We found a lower level of conjugated bilirubin at the time of surgery among PBA and a lower degree of liver fibrosis according to the Ishak score. We also found a positive correlation between fibrosis score and postnatal and corrected age at KPE with lower fibrosis values even in patients with more days of life. These data led us to confirm the hypothesis postulated by Durkin et al. that prematurity-related hepatic immaturity may offer some sort of protection against cholestatic damage.

The latter could be responsible for the initial slower trend in bilirubin clearance in PBA compared with TBA. This gap is completely recovered at 6 months after KPE (Figure 2).

Congenital anomalies are more frequently observed in preterm infants compared with full-term newborns. In our study, we reported only a higher rate of isolated cardiac malformations in the PBA cohort without increased mortality. Our results contrast with the results of Aldeiri et al., and this might be explained by the prevalence of minor cardiac malformations such as atrial and ventricular septal defects, without signs of cardiac failure (33).

Interestingly, our study shows a similar overall and 5-year transplant-free survival in PBA patients in contrast to what was reported in two similar studies where poorer outcomes in terms of COJ and NLS were observed (11, 29). According to these preliminary data, we postulated that preterm infants have less cholestatic damage and a lower degree of liver fibrosis for the same age at KPE.

Several limitations are present in this retrospective study. First, our study had a small number of patients in over 20 years of study time, during which both pre- and postoperative protocols underwent several significant changes. Second, all patients were “moderate to late” preterm in which the effect of possible confounding factors such as sepsis, delayed enteral feeding, the use of prolonged TPN, and the immaturity of liver function is less significant compared with very preterm infants.

## Conclusions

Both the PBA and TBA groups appear to have similar outcomes in terms of COJ, overall native liver survival, and 5-year liver survival.

Considering the corrected GA, early KPE is related to lower cholestatic damage. This reflects the importance of an early evaluation of preterm infants with cholestasis and the necessity for a centralization policy of BA in referral centers.

Further multicenter studies are required to enroll a larger number of patients with this rare association to confirm our preliminary data.

## Data Availability

The raw data supporting the conclusions of this article will be made available by the authors, without undue reservation.
